# *In Vivo* Multimodal Magnetic Resonance Imaging Changes After *N*-Methyl-d-Aspartate-Triggered Spasms in Infant Rats

**DOI:** 10.3389/fneur.2018.00248

**Published:** 2018-04-16

**Authors:** Minyoung Lee, Mi-Sun Yum, Dong-Cheol Woo, Woo-Hyun Shim, Tae-Sung Ko, Libor Velíšek

**Affiliations:** ^1^Department of Pediatrics, University of Ulsan College of Medicine, Ulsan, South Korea; ^2^Asan Institute for Life Sciences, Asan Medical Center, Seoul, South Korea; ^3^Department of Radiology, University of Ulsan College of Medicine, Ulsan, South Korea; ^4^Department of Cell Biology and Anatomy, New York Medical College, Valhalla, NY, United States; ^5^Department of Pediatrics, New York Medical College, Valhalla, NY, United States; ^6^Department of Neurology, New York Medical College, Valhalla, NY, United States

**Keywords:** infantile spasms, MR spectroscopy, neurometabolites, cingulate cortex, animal models

## Abstract

**Objective:**

Despite the serious neurodevelopmental sequelae of epileptic encephalopathy during infancy, the pathomechanisms involved remain unclear. To find potential biomarkers that can reflect the pathogenesis of epileptic encephalopathy, we explored the neurometabolic and microstructural sequelae after infantile spasms using a rat model of infantile spasms and *in vivo* magnetic resonance imaging techniques.

**Methods:**

Rats prenatally exposed to betamethasone were subjected to three rounds of intraperitoneal *N*-methyl-d-aspartate (NMDA) triggering of spasms or received saline injections (controls) on postnatal days (P) 12, 13, and 15. Chemical exchange saturation transfer imaging of glutamate (GluCEST) were performed at P15 and 22 and diffusion tensor imaging and additional spectroscopy (1H-MRI/MRS) of the cingulate cortex were serially done at P16, 23, and 30 and analyzed. Pathological analysis and western blotting were performed with rats sacrificed on P35.

**Results:**

Within 24 h of the three rounds of NMDA-induced spasms, there was an acute increase in the GluCEST (%) in the cortex, hippocampus, and striatum. When focused on the cingulate cortex, mean diffusivity (MD) was significantly decreased during the acute period after multiple spasms with an increase in γ-aminobutyric acid (GABA), glutamate, and glutamine *N*-acetylaspartate-plus-*N*-acetylaspartylglutamate (tNAA), total choline, and total creatine. The juvenile rats also showed decreased MD on diffusion tensor imaging and significant decreases in taurine, tNAA, and macromolecules-plus-lipids in the cingulate cortex. Pathologically, there was a significant reduction in glial fibrillary acidic protein, myelin basic protein, and neuronal nuclei expression in the cingulate cortex of rats with NMDA-induced spasms.

**Significance:**

These neurometabolic and microstructural alterations after NMDA-triggered spasms might be potential imaging biomarkers of epileptic encephalopathy.

## Introduction

Infantile spasms are an epileptic encephalopathy that requires urgent therapeutic intervention to avoid severe consequences, such as mental retardation and developmental regression (1). Diverse etiologies, including both unidentified causes and established brain pathologies, can lead to infantile spasms with diffuse electrographic abnormalities (2), which probably impair common developmental processes such as synaptogenesis, myelination, and neuronal migration, causing mental retardation or refractory epilepsies in later life (3, 4). However, these neurodevelopmental consequences have not been clearly characterized (2).

To support the rationale for an aggressive treatment approach for infantile spasms, it is crucial to determine the changes produced by the spasms in the immature brain and assess the possibility for a restoration or reversal of these changes. In children with infantile spasms, evaluation of the effects of spasms on the brain is extremely complicated because of ethical problems and a relatively small number of patients with heterogeneous etiologies (5) and treatment protocols (6, 7).

From the bench side, it would be advantageous to have a model without pre-existing lesions and a model with easily operable spasms to identify the pathological effects of the experimental spasms on the brain. The rat model of *N*-methyl-d-aspartate (NMDA)-triggered spasms (4) after prenatal betamethasone priming is regarded as having a cryptogenic etiology and the spasms are reliably triggered by the injection of NMDA. The NMDA-triggered spasms can be provoked at postnatal day 12–15, a period in rodents relevant to human infancy (3, 4). The model also displays several behavioral changes after multiple spasms (3), corresponding to those of patients with infantile spasms, and has recently been independently verified (8).

In the present study, using this rat model of NMDA-induced infantile spasms, we performed MR imaging to evaluate the acute and chronic neurometabolic and microstructural changes after spasms as potential biomarkers of epileptic encephalopathy. To add longitudinal *in vivo* data to this model, we adopted the latest high-field proton MRI technologies including chemical exchange saturation transfer imaging of glutamate (GluCEST), diffusion tensor imaging, and spectroscopy (^1^H-MRI/MRS). GluCEST imaging can map the level of glutamate *in vivo* at a high spatial resolution (9–11), and diffusion tensor imaging is used to reveal the *in vivo* connectivity of the nervous system (12, 13). ^1^H-MRS allows noninvasive quantification of brain metabolites in specific brain areas and has been successfully used in the field of epilepsy research (14, 15). Specifically, the *in vivo* developmental changes in neurometabolites and connectivity were measured in the cingulate cortex, which has extensive connections with critical regions such as the basal ganglia, thalamus, brainstem, and hippocampus (16, 17). Additional histopathologic analyses and behavioral tests were performed to further elucidate the microstructural and neurochemical changes.

## Materials and Methods

### Animals

Experiments were approved by the Institutional Animal Care and Use Committee of the Ulsan University College of Medicine and conformed to the Revised Guide for the Care and Use of Laboratory Animals [NIH GUIDE, 25(28), 1996] (18). Timed-pregnant Sprague-Dawley rats were purchased from an approved source (Orient Bio Inc., Seoul, Korea). The rats were housed individually in the animal facility during the remainder of their pregnancy with free access to standard rat chow and water on a regular 12-h light-dark cycle with the lights on at 08:00. On gestational day 15, pregnant rats received two injections of 0.4 mg/kg betamethasone (Sigma-Aldrich, St. Louis, MO, USA) at 08:30 and 18:30. Delivery occurred consistently on gestational day 22, which was considered postnatal day (P) 0 for the offspring.

**Figure 1:**
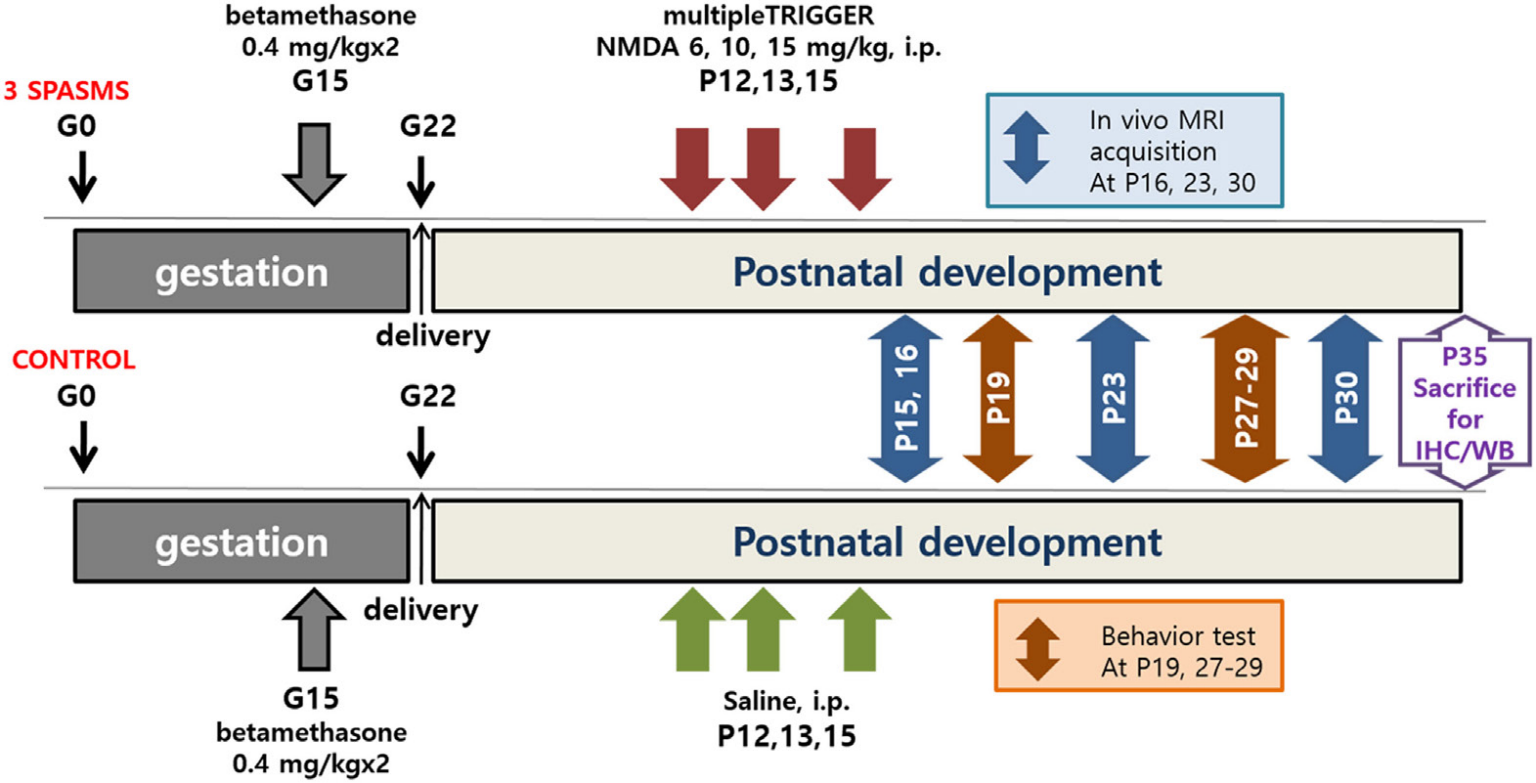
Timeline of *N*-methyl-d-aspartate (NMDA)-induced spasms, MRI acquisition, behavioral tests, and tissue collection. IHC, immunohistochemistry; WB, western blot.

Spasms were triggered by intraperitoneal injection of NMDA on P12 (6 mg/kg), P13 (10 mg/kg), and P15 (15 mg/kg); control groups received the same volume of saline (Figure 1). Immediately after NMDA administration, the rats were observed for 90 min and only the animals confirmed to have had three bouts of spasms at the appropriate time points on each day (i.e., on P12, P13, and P15) were included in the analyses (4).

### MR Imaging Studies

Animals were maintained under anesthesia with 1% isoflurane in a 1:2 mixture of O_2_:N_2_O with monitoring of their respiratory rate, electrocardiogram, and rectal temperature. GluCEST MR imaging was performed using a 7.0 T/160-mm small-animal imaging system (Bruker Pharmascan, Ettlingen, Germany) with a single-channel surface coil. Images were obtained using a 9.4 T/160-mm bore animal MRI system (Agilent Technologies, Santa Clara, CA, USA) for ^1^H-MRS and diffusion tensor imaging. Radiofrequency excitation and signal detection were accomplished with a 72-mm quadrature volume coil and a two-channel phased-array coil, respectively. Axial slices corresponding to coronal images in the neuroanatomic axis were collected from the cervical spinal cord to the olfactory bulb.

GluCEST images were acquired from an axial slice (1-mm thick) that included the hippocampal region. GluCEST images were acquired using T_2_-weighted imaging (rapid acquisition with relaxation enhancement [RARE]) with a frequency selective saturation preparation pulse comprised a Gaussian pulse with a total duration of 1,000 ms (irradiation offset of 500.0 Hz and interpulse delay of 10 µs) at a B_1_ peak of 5.6 µT. Z-spectra were obtained from −5.0 ppm to +5.0 ppm with intervals of 0.33 ppm (total, 31 images, Figure S1 in Supplementary Material). The sequence parameters were as follows: repetition time/echo time (TR/TE) = 4,200/36.4 ms, field of view = 30 mm × 30 mm, slice thickness = 1 mm, matrix size = 96 × 96, RARE factor = 16, echo spacing = 6.066 ms, and average = 1. To confirm the linear GluCEST effect, a phantom consisting of test tubes with different concentrations of glutamate (pH 7.0) images at 7 T was also done (Figure S2 in Supplementary Material).

To measure the GluCEST value (%), each region of interest (cortex, hippocampus, striatum) was drawn manually on the T_2_-weighted anatomical MR images without a frequency selective saturation preparation pulse, and the regions of interest were overlaid on the GluCEST maps. GluCEST contrast is measured as the asymmetry between an image obtained with saturation at the resonant frequency of exchangeable amine protons (+3 ppm downfield from water for glutamate) and an image with saturation equidistant upfield from water (–3 ppm), according to the following equation:

$$\text{GluCEST}(\%)=\frac{S_{-3.0\text{ ppm}}-S_{+3.0\text{ ppm}}}{S_{-3.0\text{ ppm}}}*100$$

where *S*_−3.0ppm_ and *S*_+3.0ppm_ are the magnetizations obtained with saturation at a specified offset from the water resonance of 4.7 ppm.

The B0/B1 maps on the same slices were acquired for B_0_ and B_1_ correction. The B_0_ map was calculated by linearly fitting the accumulated phase per pixel following phase unwrapping against the echo time differences from gradient echo images collected at TEs of =1.9 and 2.6 ms. B_1_ maps were calculated by using the double-angle method (flip angles 30° and 60°) and the linear correction for B_1_ was calculated as the ratio of the actual B_1_ to the expected value.

^1^H-MRI/MRS images were obtained at the assigned times as follows (Figure 1): (1) 1 day after the last cluster of spasms (P16), (2) about 1 week after the spasms (P23), and (3) about 2 weeks after the spasms (P30). The MR spectra were acquired through a signal voxel (from bregma to −3.0 mm in a coronal section, 3 mm × 2 mm × 1.5 mm) in the cingulate cortex using a point-resolved spectroscopy (PRESS) sequence for 128 acquisitions with TR/TE = 5,000/13.4 ms. For quantification, unsuppressed water signals were also acquired from the same voxel (average = 8). All the MR spectra were processed with the linear combination analysis method (LC Model ver. 6.0, Los Angeles, CA, USA) to calculate the metabolite concentrations from a fit to the experimental spectrum, based on a simulated basis set. The following brain metabolites were included in the metabolite basis set: alanine (Ala), aspartate (Asp), creatine (Cr), γ-aminobutyric acid (GABA), glucose (Glc), glutamate (Glu), glutamine (Gln), glycerophosphorylcholine (GPC), phosphorylcholine (PCh), myo-inositol (mIns), lactate (Lac), phosphocreatine (PCr), *N*-acetylaspartate (NAA), *N*-acetylaspartylglutamate (NAAG), taurine (Tau), macromolecules (MMs), and lipids (Lip). The water-suppressed time domain data were analyzed between 0.2 and 4.0 ppm, without further T1 or T2 correction. Absolute metabolite concentrations (mmol/kg wet weight) were calculated using the unsuppressed water signal as an internal reference (assuming 80% brain water content) (19). The *in vivo* proton spectra were judged to have an acceptable value if the standard deviation of the fit for the metabolite was less than 20% (Cramer–Rao lower bounds). MR diffusion tensor images were acquired using a four-shot DT-echo planar imaging sequence (TR = 3.7 s, TE = 20 ms, B0 = 1,000 s/mm^2^) with a 10-ms interval (Δ) between the application of diffusion gradient pulses, a 4-ms diffusion gradient duration (δ), a gradient amplitude (G) of 46.52 mT/m, and the Jones 30 gradient scheme.

Postprocessing analysis was performed using Diffusion Toolkit software (http://trackvis.org/). The cingulate cortex of each rat was selected and the fractional anisotropy (FA) and mean diffusivity (MD) were calculated from the diffusion tensor parametric maps. Subsequently, repeated measures ANOVA and *t*-tests were conducted to test for the treatment effect of the different diffusion parameters.

### Behavioral Testing

After NMDA-induced spasms, rats performed an open-field test on P19 and fear conditioning tests on P27–29 (Figure 1). Behavioral experiments were conducted in a standard behavioral testing room during the light phase (08:00–20:00 h) of a regular 12-h light–dark cycle.

### Open-Field Test

The locomotive activity of controls and rats after NMDA-triggered spasms was assessed using an open-field test as previously described (20). Rats were placed into a black plastic box (60 cm × 60 cm^2^ field with a 30-cm high perimeter) for 5 min, and their activities were monitored (3, 21) using computerized motion-tracking software (SMART 3.0; Panlab S.L.U., Barcelona, Spain). The center was defined as the middle area 10 cm apart from each wall and the other area was defined as the periphery (i.e., 2,000 cm^2^ periphery and 1,600 cm^2^ center; allocation of the area = 5:4).

### Fear Conditioning

The observation chamber (25 cm × 25 cm × 25 cm; Panlab s.l.u.) was constructed of aluminum (two side walls and ceiling) and Plexiglas (rear wall and hinged front door) and was situated in a soundproof box. The floor of the chamber consisted of 19 stainless steel rods (4 mm diameter) spaced 1.6 cm apart (center to center), which were connected to a shock generator and grid scrambler (Panlab s.l.u.). A tone (conditioned stimulus) was delivered by a speaker mounted on one side panel of the chamber and both the shock and tone deliveries were controlled by a computerized system.

Tone conditioning was conducted on P27. Modified from a previous report (22), the conditioning consisted of a 3-min baseline habituation in the experimental chambers, followed by exposure to five pairings of a tone (conditioned stimulus; 30 s, 85 dB, 2,000 Hz) with white-light illumination, each ending with a final footshock (unconditioned stimulus; 0.8 mA, 2.0 s) followed by a 20-s silent interval. Response to the context was monitored on P28 (24 h after the conditioning trial). The rats were returned to the chamber for 5 min, and freezing behavior was measured in response to the context. Fear response to the conditioned stimulus was conducted at 24 h after the contextual fear testing (P29). To reduce the influence of context on cued fear conditioning, tactile and visual cues were manipulated with the replacement of a wall and a floor. Following a 2-min period without a conditioned stimulus (first session, unconditioned), the rats were presented with five tone parings (85 dB, 2 kHz, 30 s, session Nos. 2, 4, 6, 8, and 10; 20 s between tone presentations, session Nos. 3, 5, 7, 9, and 11) without any foot shock. The behaviors of the rats were recorded and analyzed using the signal generated by a high-sensitivity weight transducer system (Panlab s.l.u.).

### Sample Preparation and Immunohistochemistry

The animals with three bouts of spasms on P12, P13, and P15 and the corresponding controls were transcardially perfused with 4% paraformaldehyde on P35 under deep anesthesia, and their brains were removed and cryoprotected. Twenty-micron serial coronal sections were cut on a cryocut microtome.

For immunohistochemistry, myelin basic protein (MBP) antibody (1:1,000; Santa Cruz Biotechnology, Inc., Santa Cruz, CA, USA), glial fibrillary acidic protein (GFAP) antibody (1:2,000; EMD Millipore, Billerica, MA, USA), or neuronal nuclei (NeuN) antibody (1:2,000; EMD Millipore) were used as primary antibodies. All slides were examined by light microscopy (Olympus BX-53; Olympus Co., Tokyo, Japan). Semi-quantification analysis of immunohistochemical staining was performed using ImageJ (NIH, Bethesda, MD). The corresponding area in the cingulate cortex cg2 subfield was analyzed to determine the NeuN- and GFAP-positive cell count and the stained area measurement of MBP. Three or five randomly selected areas (1.0 × 10^4^ sq μm) on each section were used for NeuN- and GFAP-positive cell counting. The same areas of binarized images of MBP-stained sections were quantified from each animal.

### Western Blot Analysis

After deep anesthesia, bilateral cortical tissues from bregma to the posterior hippocampal area without the hippocampus (anterior posterior 0 to −5 mm) were collected from controls and rats with NMDA-triggered spasms on P35. Anti-MBP (1:2,000; Santa Cruz Biotechnology, Inc.), anti-NeuN (1:5,000; EMD Millipore), or anti-GFAP (1:5,000; EMD Millipore) antibodies were used. Normalization was performed by the development of parallel western blots probed with β-actin (1:20,000; Santa Cruz Biotechnology Inc.) antibody analyzed by densitometry using ImageJ software (NIH, Bethesda, MD).

### Statistics

The Mann–Whitney’s *U* test was used for the comparison of two groups and Student’s *t*-test was used for the data following normal distribution. The concentrations of metabolites were compared between the groups and time points using a linear mixed model. The absence of significant sex-based differences (*p* always > 0.10) was confirmed in advance, and both sexes were combined. The significance level was preset to *p* < 0.05. Statistical analyses were conducted using SAS^®^ Version 9.3 (SAS Institute Inc., Cary, NC, USA) and SPSS 18.0 (IBM Corp., Armonk, NY, USA).

## Results

### Mapping of the Glutamate Concentration at Acute Periods After Three Rounds of NMDA Spasms

**Figure 2:**
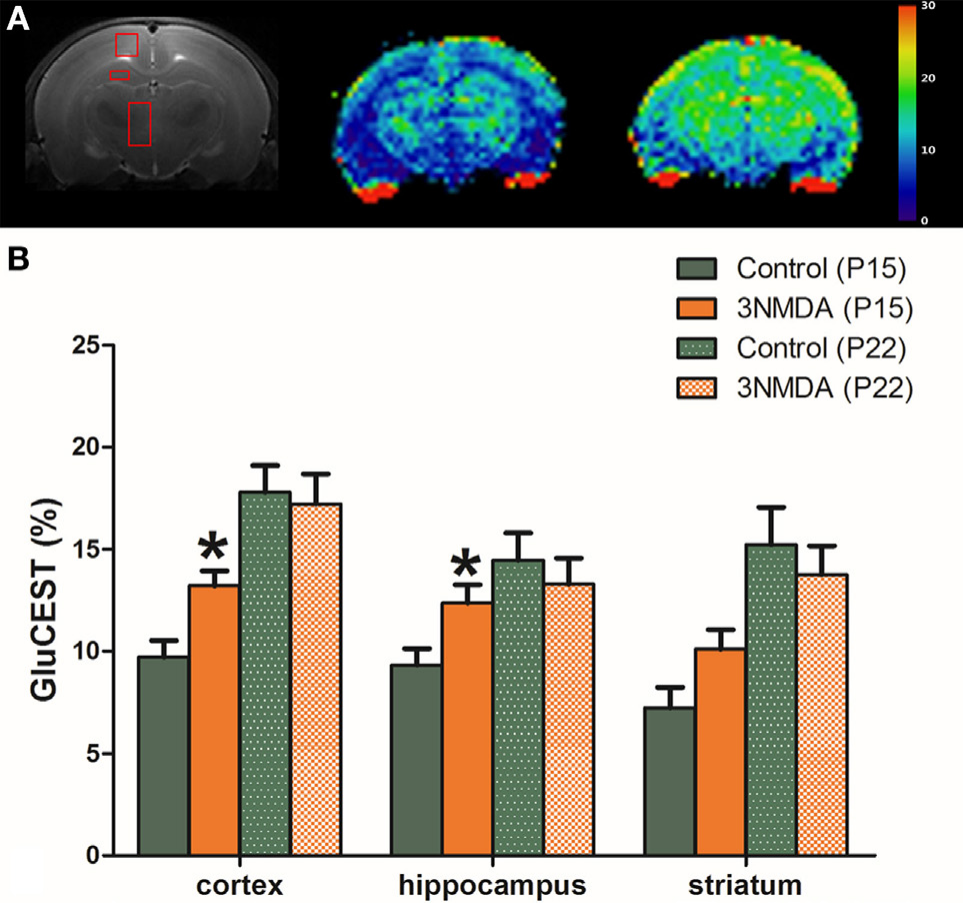
**(A)** After three rounds of spasms on postnatal day (P) 12, 13, and 15, glutamate chemical exchange saturation transfer (GluCEST, %) levels were measured in the cortex, hippocampus, and striatum on P15. **(B)** There were significant increases in the GluCEST (%) of rats with three rounds of *N*-methyl-d-aspartate (NMDA)-induced spasms compared with controls in all measured areas. A developmental increase in the GluCEST (%) from P15 to P22 was noted, and there was no significant difference in the GluCEST (%) between rats with spasms and controls.

There were significant increases in the GluCEST (%) levels of rats with three rounds of NMDA-induced spasms in the cortex and hippocampus at P15 (N = 14; cortex, 13.20 ± 0.74%, *p* = 0.011; hippocampus, 12.36 ± 0.91%, *p* = 0.016; striatum, 10.10 ± 0.96%, *p* = 0.062) compared with controls (N = 14, 9.71 ± 0.82%, 9.32 ± 0.81%, 7.23 ± 1.00% in each area). There was a developmental increase in the GluCEST (%) at P22 but no significant difference in the GluCEST (%) between the rats with spasms and controls (Figure 2).

### Neurochemical Alterations in the Cingulate Cortex After Multiple NMDA-Triggered Spasms

**Figure 3:**
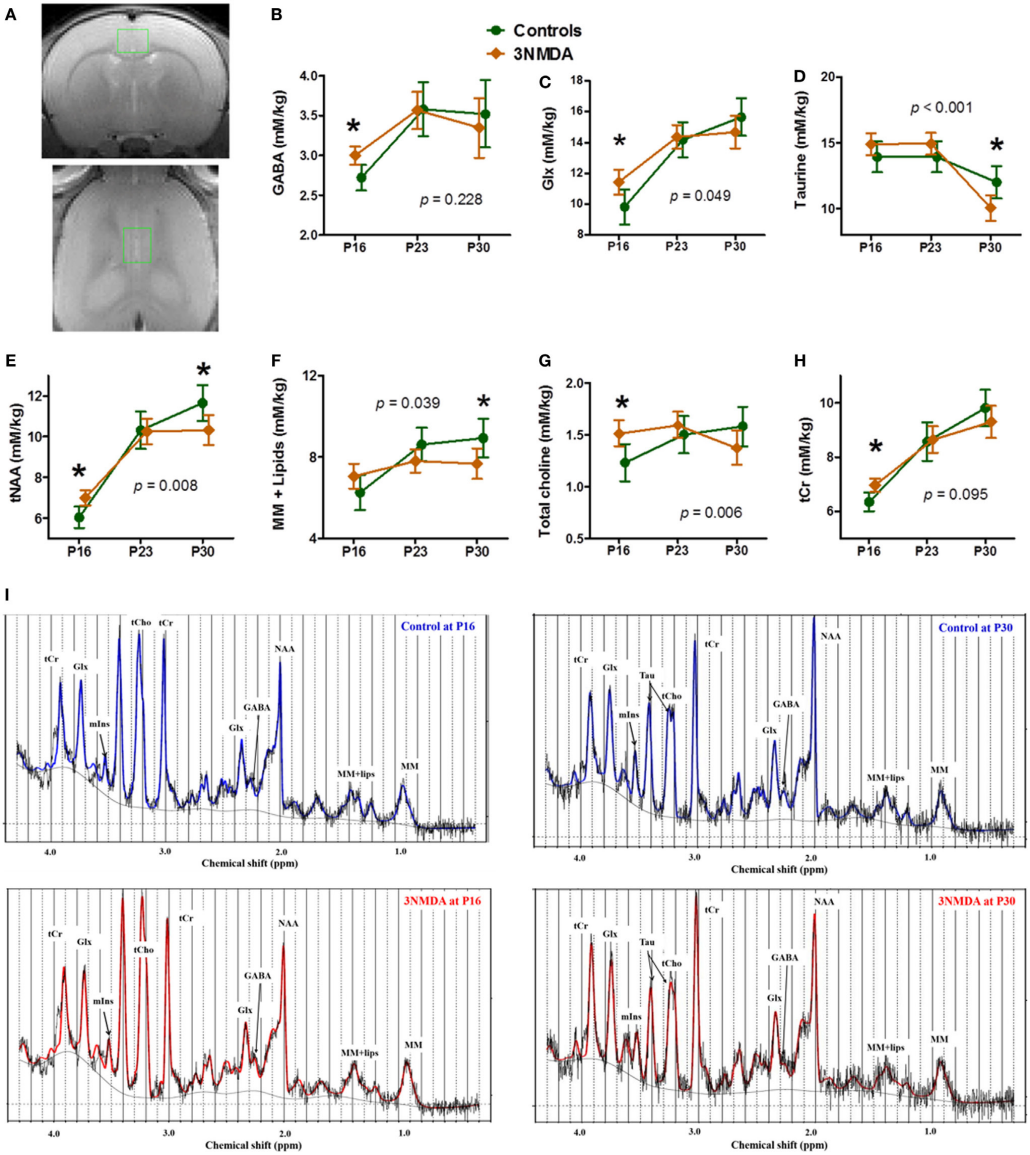
Neurochemical changes in rats with *N*-methyl-d-aspartate (NMDA)-induced spasms using ^1^H-MRS findings. **(A)** A single voxel position in the cingulate cortex (top, coronal section; bottom, horizontal section). The brain levels of GABA, glutamine + glutamate (Glx), tNAA, total choline, and tCr were significantly higher in rats with NMDA-induced spasms on P16 **(B,C,E,G,H)**. **(D–F)** At 2 weeks, taurine (Tau), tNAA, and macromolecules + lipids (MM + lipids) were significantly decreased in rats with spasms. **(C–G)** The changes in Glx, Tau, tNAA, MM + lipids, and total choline with time were significantly different between the groups. **(I)** The examples of MR spectra from rats with or without 3 NMDA spasms on each time points, P16 and P30 clearly shows the neurometabolic changes after NMDA-induced spasms. *indicates a significant difference between the groups at each time point (*p* < 0.05). Using a generalized linear mixed model, there were also significantly different interactions between the groups and time points for Glx, Tau, tNAA, MM + lipids, and total choline (each *p*-value is marked on the graph). All imaging was done on the same timepoints (P16, 23, and 30), although the data points are not aligned across groups to avoid overlapping of the two graphs.

There was no significant morphological difference between the two groups (NMDA-induced spasms [*n* = 12] and controls [*n* = 10]) on visual evaluation of serial T2-weighted MR images. One day after the last NMDA injection (P16), GABA (*p* = 0.008), Glx (Glu + Gln, *p* = 0.024), tNAA (*p* = 0.006), tCho (*p* = 0.014), and tCr (*p* = 0.007) levels measured from cingulate cortex (Figure 3A) were significantly increased in rats with NMDA-induced spasms (Figures 3B,E,G,H). At 1 week (P23) after the NMDA-induced spasms, there were also no significant differences in the concentrations of the neurochemicals measured in the cingulate cortex between the two groups. At 2 weeks (P30) after the NMDA-induced spasms, Tau (*p* = 0.016), tNAA (*p* = 0.026), and MM + Lips (*p* = 0.042) were significantly decreased compared with controls (P30; Figures 3D–F). The temporal changes in each neurometabolite were compared between the groups, and glutamine and glutamate (Glx, *F* = 3.30, DF1 = 2, DF2 = 34.7, *p* = 0.049), Tau (*F* = 10.52, DF1 = 2, DF2 = 32.5, *p* < 0.001), tNAA (NAA + NAAG, *F* = 6.57, DF1 = 2, DF2 = 15.7, *p* = 0.008), MMs and lipids (MM + Lips, *F* = 4.01, DF1 = 2, DF2 = 15.4, *p* = 0.039), and total choline (tCho, *p* = 0.006) were significantly different between the two groups (Figures 3C–G).

### In Vivo Microstructural Alterations Following Multiple Bouts of NMDA-Triggered Spasms

**Figure 4:**
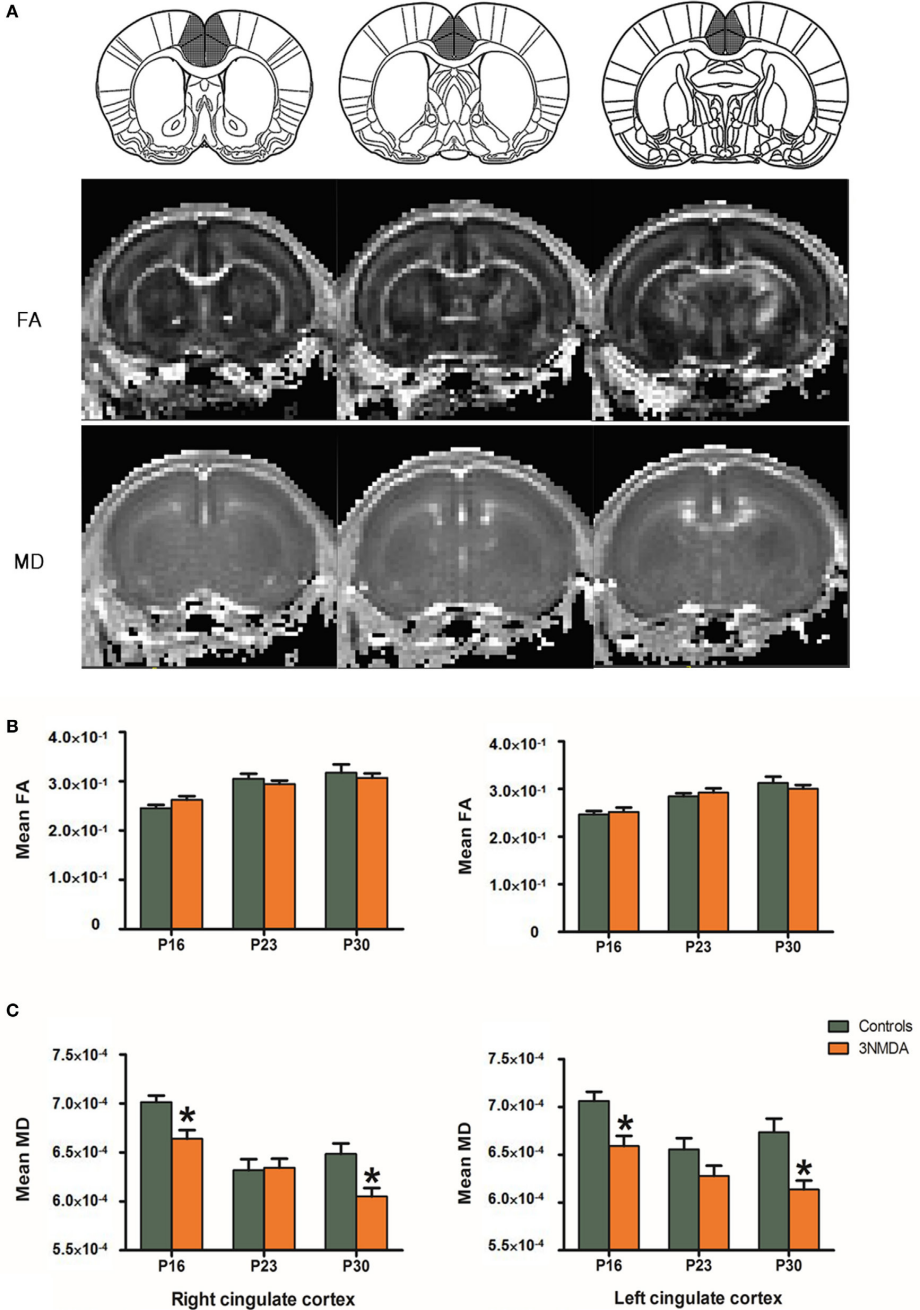
**(A)** On diffusion tensor imaging of rats after *N*-methyl-d-aspartate (NMDA)-induced spasms and controls, fractional anisotropy (FA) and mean diffusivity (MD) were measured in the cingulate cortex of each animal. **(B)** The anatomical location of cingulate cortex was clearly identified on FA/MD maps of the rat at P16. **(C)** There were no significant differences in the FA values at each time point between the rats with spasms (N = 15) and controls (N = 15). Rats with multiple bouts of NMDA-triggered spasms showed a significantly decreased MD at P16 and P30 in both cingulate cortices compared with controls.

There was no significant difference in the FA values at each time point between rats with spasms (N = 15) and controls (N = 15). The rats with multiple bouts of NMDA-triggered spasms showed a significantly decreased MD at P16 and P30 in both cingulate cortices (right/left cingulate cortex: P16, 6.64 × 10^−4^/6.59 × 10^−4^, *p* = 0.012/*p* = 0.001; P30, 6.05 × 10^−4^/6.14 × 10^−4^; *p* = 0.036/*p* = 0.016) compared with controls (P16, 7.01 × 10^−4^/7.06 × 10^−4^; P30, 6.48 × 10^−4^/6.74 × 10^−4^, Figure 4).

### Behavioral Changes After Multiple Bouts of NMDA-Triggered Spasms

**Figure 5:**
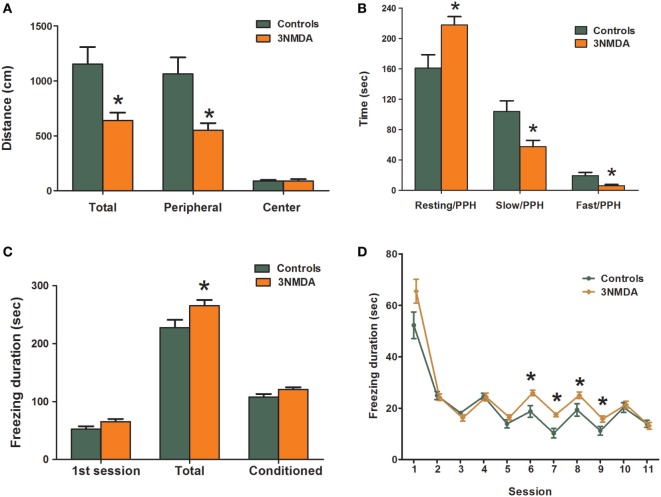
Comparison of behavioral changes between rats with *N*-methyl-d-aspartate (NMDA)-induced spasms and controls. **(A,B)** Open-field test showing significantly decreased exploration distances in rats with NMDA-induced spasms in peripheral and total areas **(A)** and significantly increased resting activities and decreased slow and fast activities in the peripheral area **(B)** of rats with spasms. The rats with NMDA-induced spasms also showed significantly increased durations of freezing to sound and light stimuli **(C)** that persisted after several sessions without shocks **(D)** in a fear conditioning test. There was a significant difference in freezing durations between the two groups. *indicates a significant difference between the groups (*p < 0.05, Mann–Whitney *U* test).

To evaluate the behavioral changes that occurred after multiple bouts of NMDA-induced spasms, the results of open-field and fear conditioning tests were compared between the rats that had experienced three bouts of NMDA-triggered spasms and controls. In the open-field test on P19, the rats with NMDA-induced spasms (*n* = 23) traveled a significantly shorter distance through the peripheral area (*p* = 0.005) as well as in the total area (*p* = 0.006) compared with controls (*n* = 20; Figure 5A). Resting time in the peripheral area was significantly increased in rats with NMDA-induced spasms (*p* = 0.012) compared with controls. The time spent in slow and fast motion in the peripheral area was significantly decreased in the rats with NMDA-induced spasms compared with controls (*p* = 0.014 and *p* = 0.010, respectively; Figure 5B). The time spent in the center was equivalent in the two groups.

In fear conditioning tests on P27–29, there was no difference in tone conditioning on P27 or response to context on P28. However, on P29, the total freezing durations were significantly increased in rats with NMDA-induced spasms compared with controls (*p* = 0.037; Figure 5C). During the first session, freezing durations were not different between the two groups (*p* = 0.058), but freezing durations were significantly increased in rats with NMDA-induced spasms at conditioned sessions from 6 to 9, in particular (*p* = 0.007, 0.002, 0.047, and 0.022, respectively; Figure 5D).

### Reduced GFAP, MBP, and NeuN Expression in the Juvenile Period After Multiple Bouts of NMDA-Triggered Spasms

**Figure 6:**
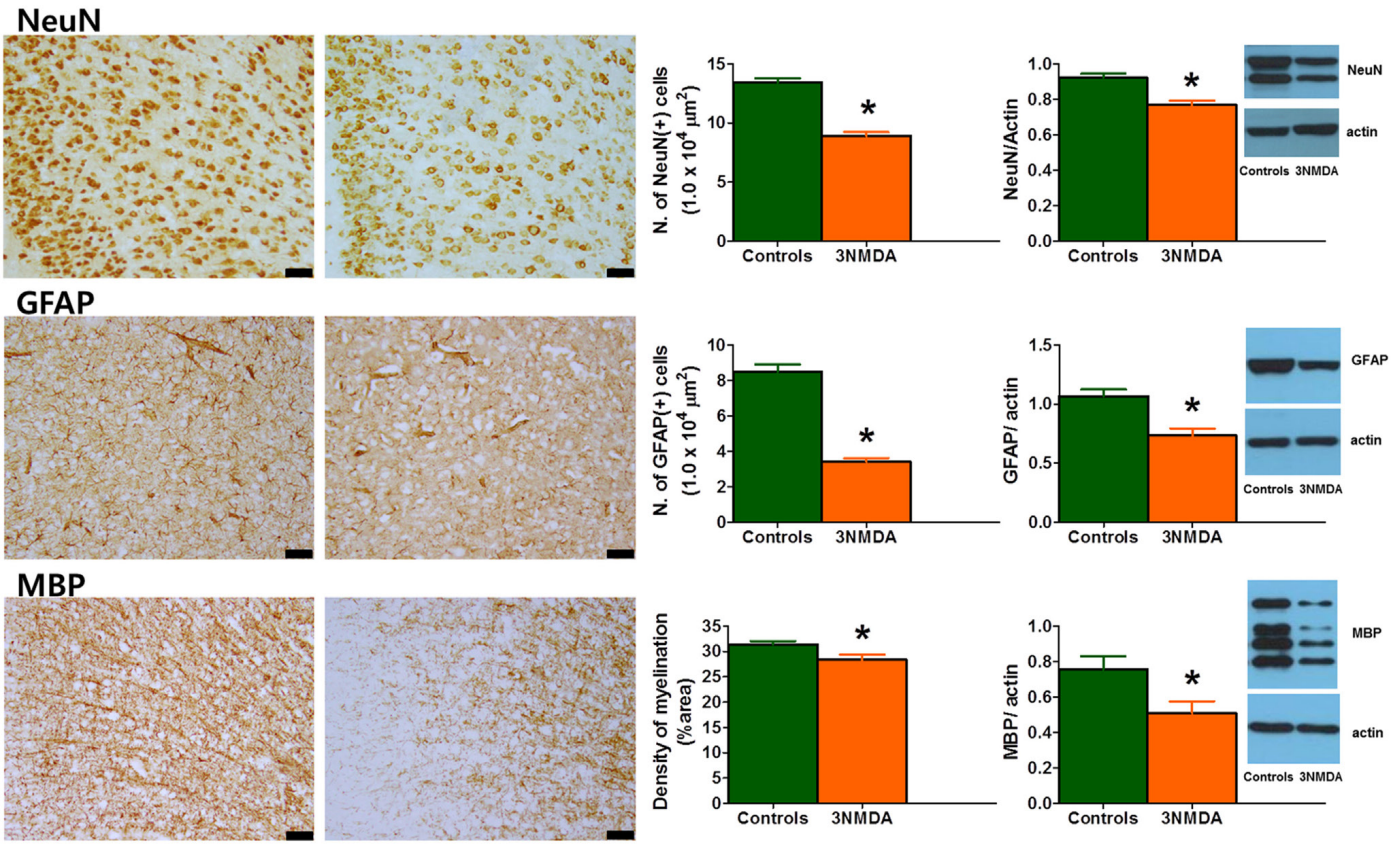
Glial fibrillary acidic protein (GFAP), myelin basic protein (MBP), and neuronal nuclei (NeuN) expression in the cingulate cortex during the juvenile period. (Left) Immunohistochemical staining of the cingulate cortex on P35 revealed decreased expression of GFAP, MBP, and NeuN in rats with three bouts of *N*-methyl-d-aspartate (NMDA)-induced spasms. The scale bars indicate 50 µm. (Right) Western blot analysis of the rat cortex on P35 showed significantly decreased expression of GFAP, MBP, and NeuN after multiple bouts of NMDA-induced spasms (*p < 0.05, Mann–Whitney *U* test).

Semi-quantitative analysis of immunohistochemical staining on P35 revealed a significant reduction in NeuN- and GFAP-positive cells (both *p* < 0.001) and MBP-positive area (*p* = 0.041) in rats with three bouts of NMDA-triggered spasms (on P12, P13, and P15, *n* = 13) compared with controls (*n* = 11) (Figure 6). The cortical protein expression levels of GFAP, MBP, and NeuN on P35 were quantified and compared between the rats with multiple spasms and controls. GFAP (*p* = 0.007), MBP (*p* = 0.027), and NeuN (*p* = 0.001) expression was significantly reduced in the cortex of rats with NMDA-induced spasms (*n* = 13) compared with controls (*n* = 13; Figure 6).

## Discussion

An early diagnosis of infantile spasms has been considered critical for improving the neurodevelopmental outcomes of affected patients (23). However, it is uncertain which part of the developing brain is affected by infantile spasms, as is the extent of any brain insult and whether it is reversible. Our present study focused on metabolic and microstructural brain insults after spasms in a rat model of infantile spasms using *in vivo* MR imaging. Behavioral tests and *ex vivo* histopathological processes were employed to support the results.

Considering the increased metabolism of brain tissue during seizures, neurochemical dysfunction might be the main pathophysiologic feature of the epileptic brain (24), especially during the acute stage. The GluCEST technique quantifies glutamate by measuring proton exchange between the amine protons of the glutamate and the water protons and the measurement is at least 100 times more sensitive than the traditional 1H MRS method (9, 25). The GluCEST is suggested as a surrogate marker of glutamate concentration in glial cells at the synaptic level (25) and glutamate contributes >90% of the GluCEST signal with <10% contribution from other metabolites in glial neuronal unit (10). Previous study with temporal lobe epilepsy patients also showed the high potential of GluCEST identifying the epileptogenic foci (26). Thus, we hypothesized that the area of hyperexcitation in rats with NMDA-induced spasms can be screened with GluCEST imaging with high spatial resolution during acute periods. GluCEST mapping showed significantly increased glutamate in cortex and hippocampus at P15 after multiple rounds of spasms (Figure 2), which suggests that the cortex is one of the major brain area of hyperexcitation by the NMDA-induced spasms.

Our *in vivo* serial neurochemical/microstructural analysis focused on the cingulate cortex in the rat after multiple bouts of spasms. The cingulate cortex is a region of the limbic system reciprocally linked to the hippocampus (17) and with extensive connections to the critical regions for spasm generation, including the basal ganglia, thalamus, and brainstem (16, 27). In the cingulate cortex, consistent with the findings of GluCEST imaging, acute elevations in GABA, Glx, tNAA, tCho, and tCr were noted in rats after multiple bouts of NMDA-induced spasms compared with controls (Figure 3). The glutamate plus glutamine levels likely represent the neuronal metabolic pool. *In vivo* human proton MRS studies (28, 29) have also reported alterations in GABA and glutamate in patients with epilepsy. In addition, choline (Cho) represents a precursor for the neurotransmitter acetylcholine (30) and creatinine is associated with energy metabolism (31, 32). The early elevation of markers of neuronal metabolism together with the excitatory neurotransmitters in the cingulate cortex may also reflect activation of the cingulate cortex during NMDA-induced spasms in this model.

With diffusion tensor imaging, we also identified an acute decrease in MD in the cingulate cortex after three bouts of spasms, which represent the early brain damage (33). MD is an inverse measure of the membrane density, is sensitive to cellularity, edema, and necrosis (34). MD decrease quickly after injury (first day) in most models studied, which is consistent with our finding at P16. The decrease was explained by the decrease in water diffusion associated with injury such as cellular edema, despite there is still debate on the precise mechanism for the decrease in MD associated with injury. Previously postictal decrease of MD was reported in human (35), which is consistent with our finding and probably reflects neuronal swelling by excitotoxic injury in the brain areas involved in seizure.

In addition to these acute changes associated with spasms, *in vivo* MR imaging enables us to perform a longitudinal follow-up of neurometabolic and microstructural alterations in rats with NMDA-triggered spasms. NMDA is an excitatory amino acid that can cause neuronal death and glial activation is well observed in kainic acid animal model and tissues of patients with chronic epilepsy (36). However, gross morphological changes caused by NMDA-induced spasms in our model were not evident on brain MR imaging and a previous pathologic report of NMDA-induced seizures in young animals also reported no gross morphological changes on pathology (37). Consistently with this finding, FA, a gross measure of microstructural integrity marker that is less specific to the type of injury (33), is not affected in every time point of this study.

In the cingulate cortices of the juvenile rats with NMDA-induced spasms (P30), tNAA, Tau, and MM + lipids were significantly decreased with a significant reduction in MD. We also found decreased expression of neuronal, astrocyte, and myelination markers after NMDA-induced spasms in these rats. These findings suggest the different mechanism of injury during infancy compared to that of the adulthood. Instead of cell death or gliosis after excitatory injury, the developmental process of their brain, active protein synthesis and cell proliferation, may be involved. These pathologic changes are also in line with a significant reduction in neuronal markers, NAA, and Tau and markers of myelin production and substructural components such as MMs and lipids (31, 38), as well as the reduced MD reflecting decreased membrane density and cellularity (34, 39).

Previous research into epilepsy has already shown the typical developmental pattern of neurometabolites in both a rodent model (40) and in humans (32). NMDA-triggered spasms significantly impaired this time-dependent neurometabolic developmental pattern of Glx, Tau, tNAA, MM + lipids, and total choline (Figure 3). These combined alterations in neurometabolites and structural maturation markers in this animal model indicate the compromised development of the cingulate cortex following NMDA-triggered spasms. Also, the decrease of all proteins examined can be explained by a decrease in protein synthesis during the critical brain development after NMDA-triggered spasms.

Furthermore, decreased exploratory behaviors in the peripheral areas and increased freezing activities to conditioned sound and light stimuli were also observed in our study group that suffered from NMDA-induced spasms. Other behavioral disruptions were found in previous studies with similar models (21, 37). Considering the widespread reduction in neuronal metabolites in neurodevelopmental disorders (31) and a positive correlation between motor skill learning and myelination (41), the pathological changes in the brain might explain these behavioral changes.

In conclusion, NMDA-induced spasms during infancy lead to time-dependent neurochemical and microstructural changes in the cingulate cortex and subsequent pathologic changes during the juvenile period. These age-dependent alterations in neurometabolites after NMDA-triggered spasms should be further explored as potential biomarkers of outcomes in human infantile spasms or other epileptic encephalopathies.

## Ethics Statement

We confirm that we have read the Journal’s position on issues involved in ethical publication and affirm that this report is consistent with those guidelines.

## Author Contributions

ML: manuscript writing, experiments, and statistics; MSY: main idea, manuscript writing and revision, and experiments guiding; DCW: manuscript revision and GluCEST analysis; WHS: DTI, FA, and MD analysis; TSK: manuscript revision; LV: manuscript revision.

## Conflict of Interest Statement

The authors declare that the research was conducted in the absence of any commercial or financial relationships that could be construed as a potential conflict of interest.
