# Supplementary material for: In Vivo Multimodal Magnetic Resonance Imaging Changes After N-Methyl-d-Aspartate-Triggered Spasms in Infant Rats
Source: Front Neurol. 2018 Apr 16;9:248. doi: 10.3389/fneur.2018.00248 (PMC5911983; doi:10.3389/fneur.2018.00248)
Supplement: Supplementary file 1 [file presentation_1.PDF]

Supplementary figure legends

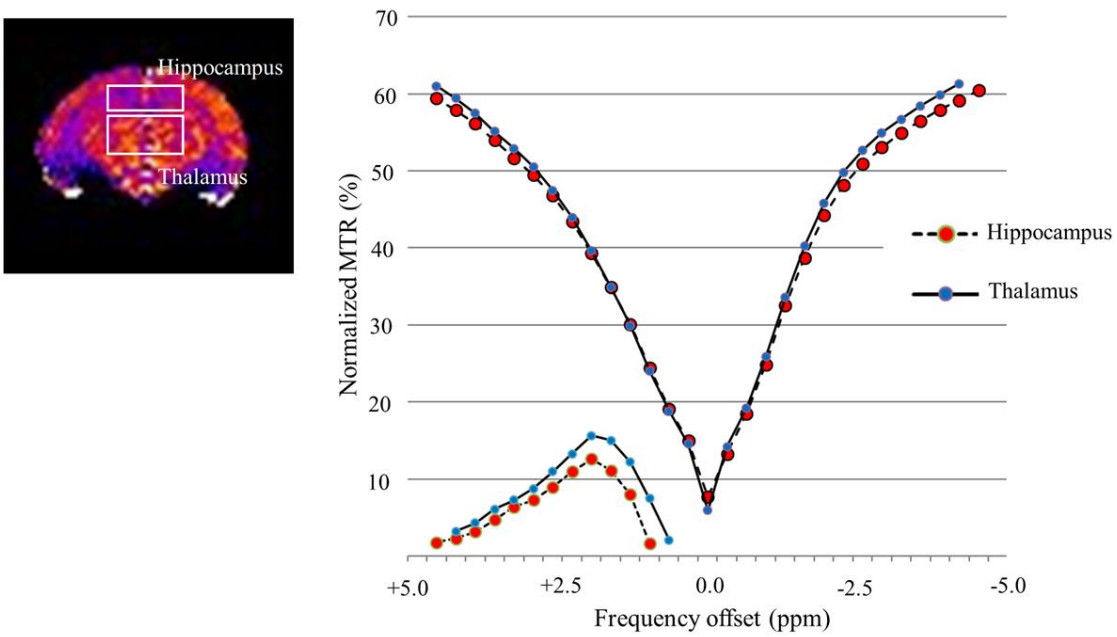

S1.

The color coded image of GluCEST with two region of interests on hippocampus and thalamus and experimentally acquired Z-spectral (Z+1400 to –1400 Hz) signal intensity. MTRasym(%) curves of each region are shown at the bottom left.

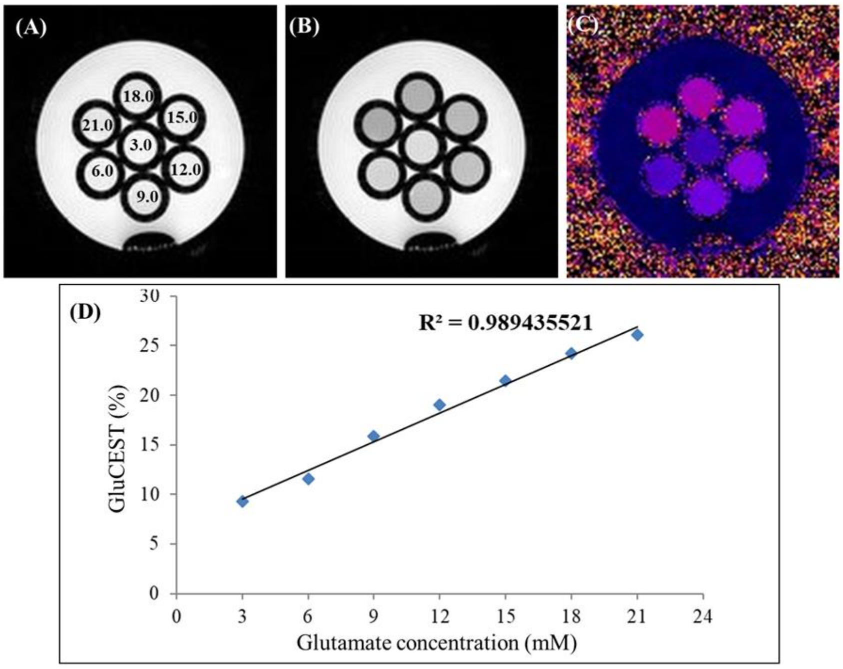

S2.

GluCEST images at 7T of a phantom were performed at 37 °C. Experimental test tubes with different concentrations of glutamate (pH 7.0) immersed in a beaker containing PBS. The numbers indicates the concentration of glutamate (mM). Two T2 weighted images were acquired with application of saturation pulse train with B1rms = 5.6 uT for 1 sec at (A) -3 ppm and (B) +3 ppm. (C) contrast color-coded image on the original CEST image. (D) Linear dependence (R2 = 0.989) of GluCEST effect on glutamate (Glu) concentration with a slope of ~0.6% per mM glutamate.
