# Supplementary material for: In Vivo Multimodal Magnetic Resonance Imaging Changes After N-Methyl-d-Aspartate-Triggered Spasms in Infant Rats
Source: Front Neurol. 2018 Apr 16;9:248. doi: 10.3389/fneur.2018.00248 (PMC5911983; doi:10.3389/fneur.2018.00248)
Supplement: Supplementary file 2 [file presentation_2.ppt]

## Slide 1
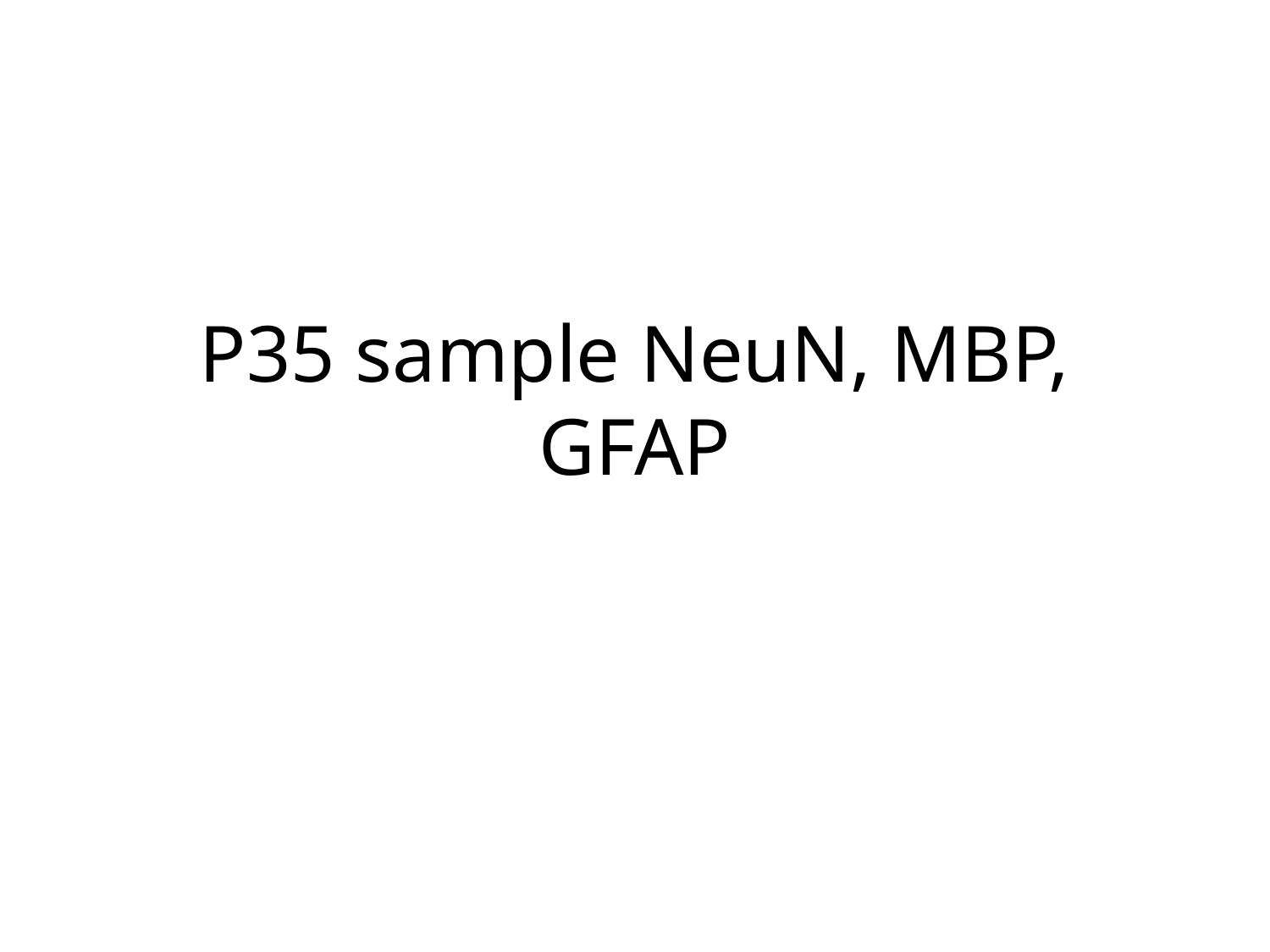

# P35 sample NeuN, MBP, GFAP

## Slide 2
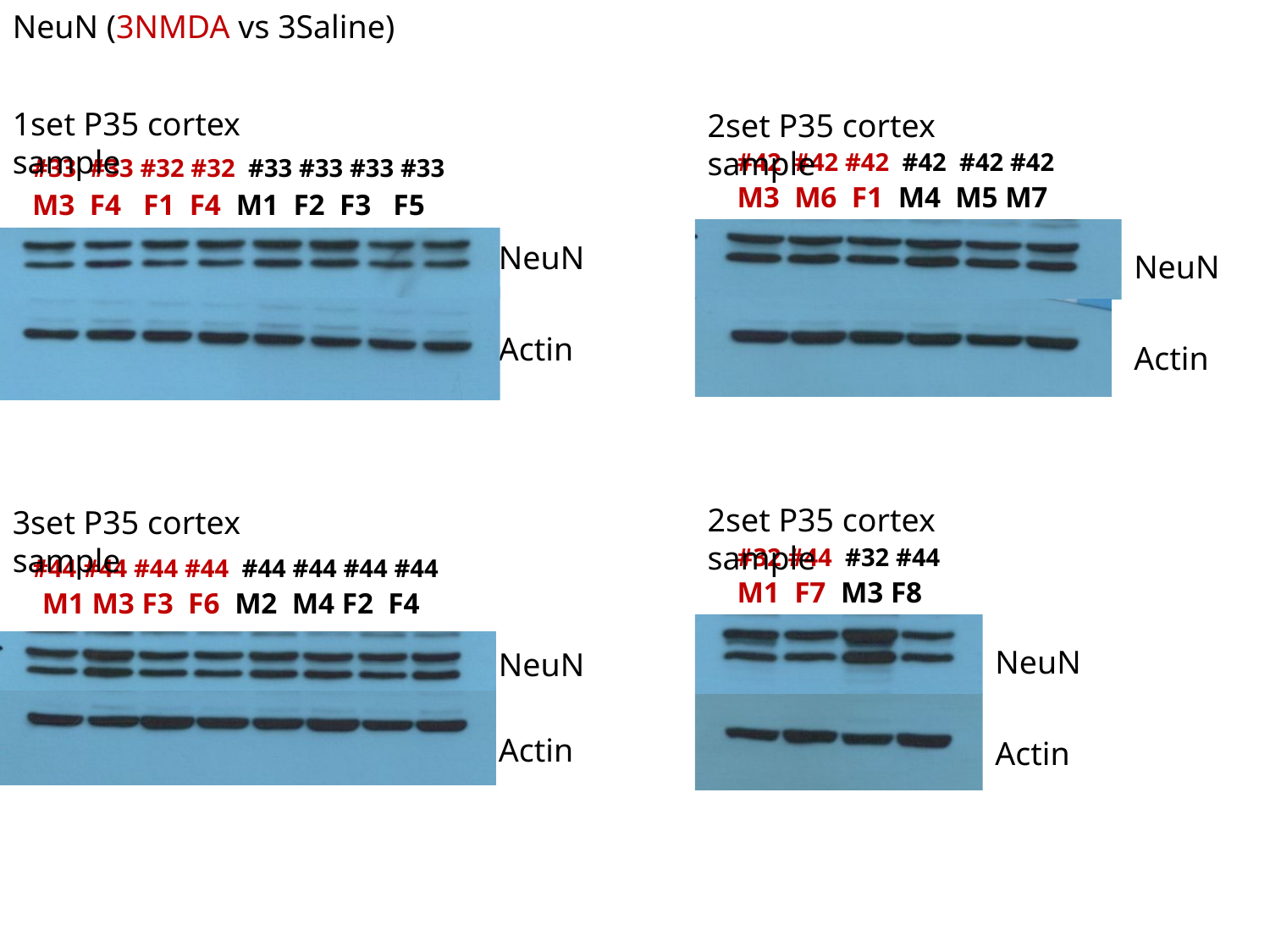

NeuN (3NMDA vs 3Saline)
1set P35 cortex sample
2set P35 cortex sample
#42 #42 #42 #42 #42 #42
#33 #33 #32 #32 #33 #33 #33 #33
M3 M6 F1 M4 M5 M7
M3 F4 F1 F4 M1 F2 F3 F5
NeuN
NeuN
Actin
Actin
2set P35 cortex sample
3set P35 cortex sample
#32 #44 #32 #44
#44 #44 #44 #44 #44 #44 #44 #44
M1 F7 M3 F8
M1 M3 F3 F6 M2 M4 F2 F4
NeuN
NeuN
Actin
Actin

## Slide 3
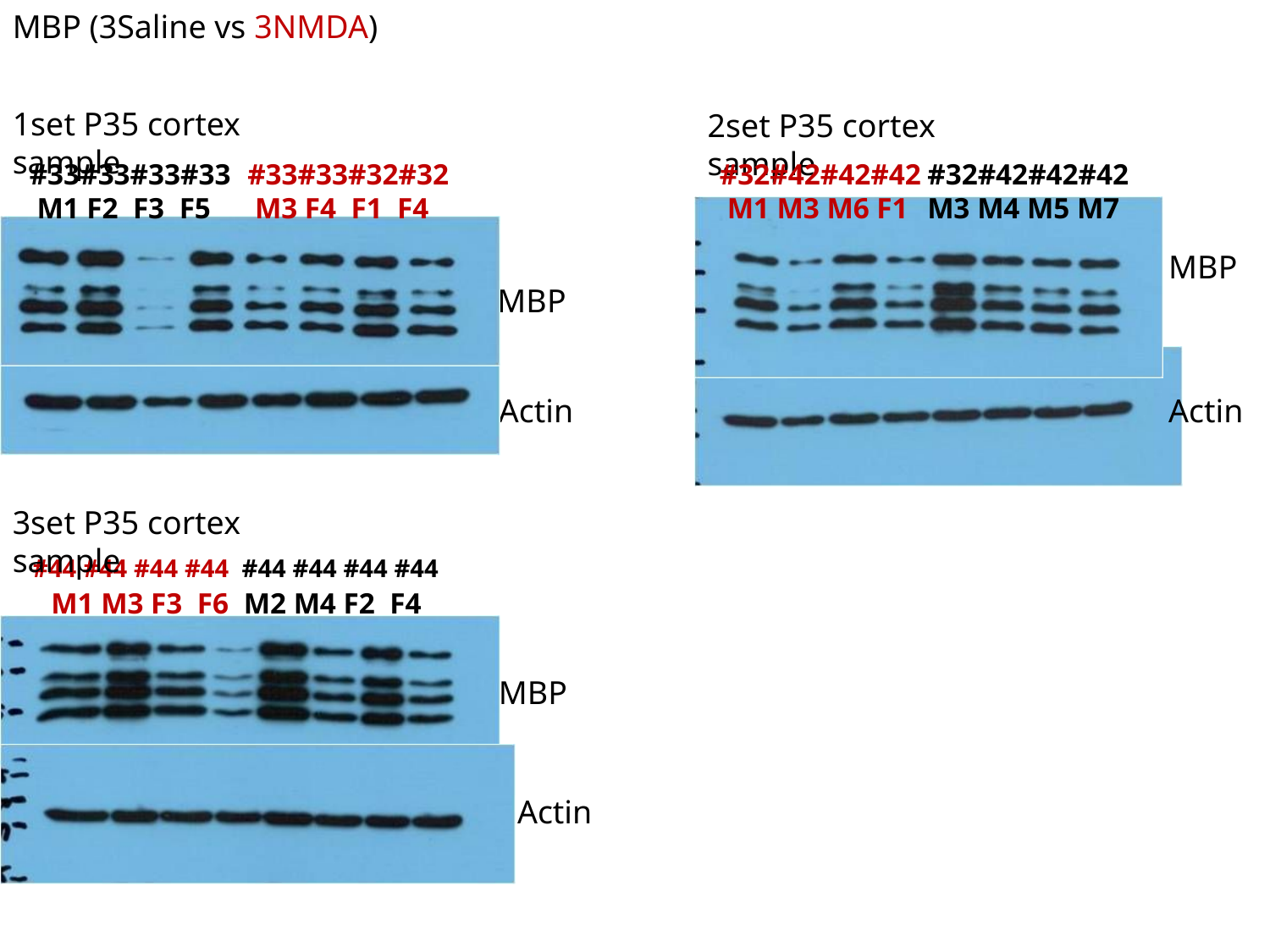

MBP (3Saline vs 3NMDA)
1set P35 cortex sample
2set P35 cortex sample
#33#33#33#33
 M1 F2 F3 F5
#33#33#32#32
 M3 F4 F1 F4
#32#42#42#42
 M1 M3 M6 F1
#32#42#42#42
M3 M4 M5 M7
MBP
MBP
Actin
Actin
3set P35 cortex sample
#44 #44 #44 #44 #44 #44 #44 #44
M1 M3 F3 F6 M2 M4 F2 F4
MBP
Actin

## Slide 4
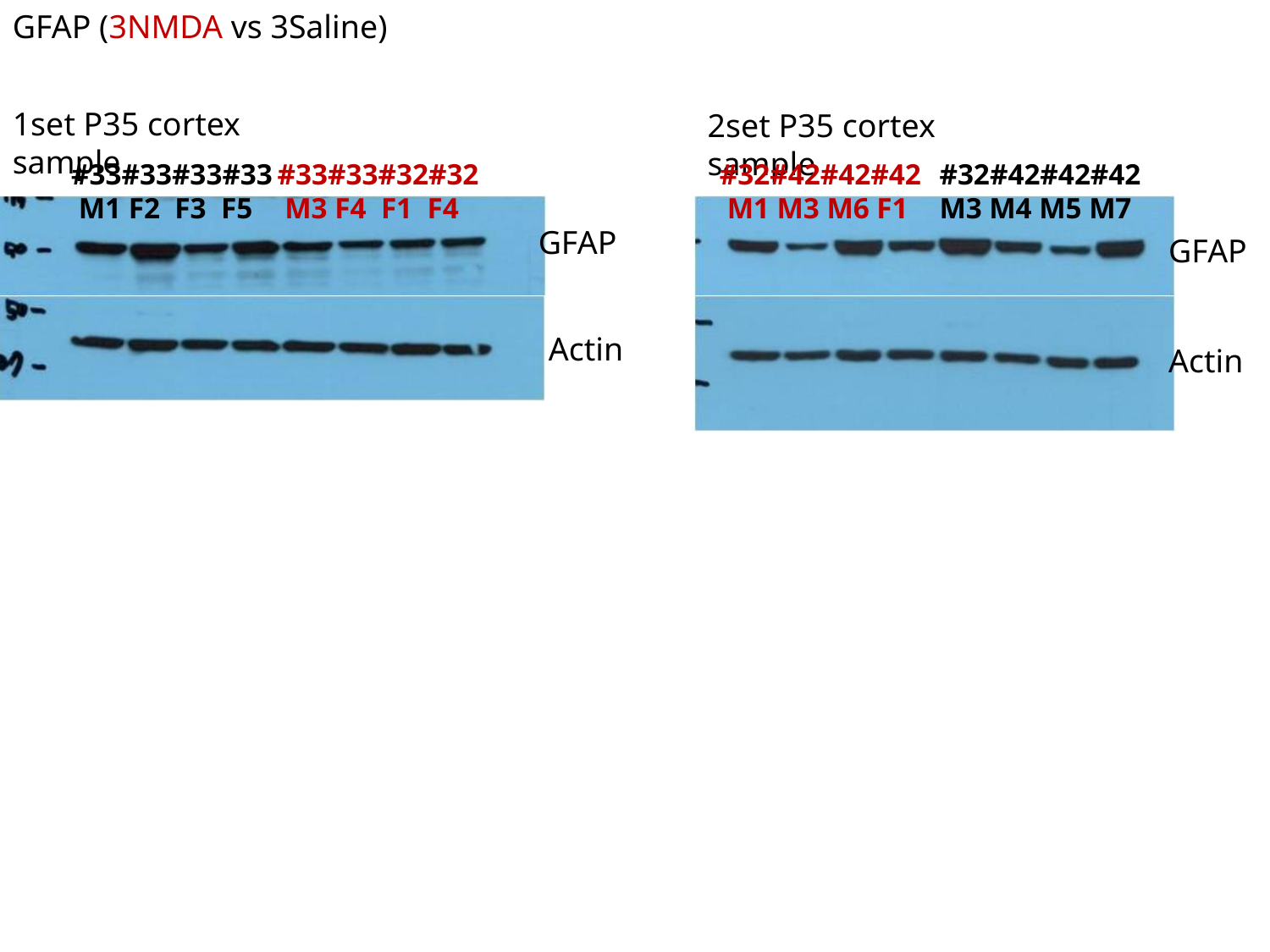

GFAP (3NMDA vs 3Saline)
1set P35 cortex sample
2set P35 cortex sample
#33#33#33#33
 M1 F2 F3 F5
#33#33#32#32
 M3 F4 F1 F4
#32#42#42#42
 M1 M3 M6 F1
#32#42#42#42
M3 M4 M5 M7
GFAP
GFAP
Actin
Actin
